# Morph-specific Dorsal Fin Characteristics and Sex Steroids in Muscles Across Life History Stages in a Sex Dynamic Marine Fish, *Lythrypnus dalli*

**DOI:** 10.1093/iob/obag035

**Published:** 2026-07-10

**Authors:** J M Starkey, K J White, R Sapkota, C J Richards, S L Barber, D S Pradhan

**Affiliations:** Department of Biological Sciences, Idaho State University, 921 S. 8th Ave, Mail Stop 8007, Pocatello, ID 83209, USA; Department of Biology and Allied Health, College of Southern Idaho, 315 Falls Ave, PO Box 1238, Twin Falls, ID 83303-1238, USA; Department of Biological Sciences, Idaho State University, 921 S. 8th Ave, Mail Stop 8007, Pocatello, ID 83209, USA; Department of Biological Sciences, Idaho State University, 921 S. 8th Ave, Mail Stop 8007, Pocatello, ID 83209, USA; Department of Biological Sciences, Idaho State University, 921 S. 8th Ave, Mail Stop 8007, Pocatello, ID 83209, USA; Department of Biological Sciences, Idaho State University, 921 S. 8th Ave, Mail Stop 8007, Pocatello, ID 83209, USA; Department of Biological Sciences, Idaho State University, 921 S. 8th Ave, Mail Stop 8007, Pocatello, ID 83209, USA; Department of Biomedical Sciences, Idaho State University, 921 S. 8th Ave, Mail Stop 8288, Pocatello, ID 83209, USA

## Abstract

While sex steroids orchestrate the organization and activation of external anatomical and behavioral phenotypes, tissue specific mechanisms that regulate these processes are largely unknown. To understand the regulation of both fixed and plastic morphological and endocrine characteristics across social contexts, we investigated the bluebanded goby, *Lythrypnus dalli*, a sexually plastic fish. The distribution and density of androgen receptors is sexually dimorphic in skeletal muscles of these fish and is associated with courtship displays in males. The supracarinalis muscle, which runs dorsal and parallel to the spinal cord is used for courtship displays by males and for aggressive displays by all *L. dalli* morphs. Here, we first investigated dorsal fin lengths across fish from different life history stages. We found that while the distribution of dorsal fin sizes exists on a continuum, 45.54% of males had dramatically longer dorsal fins compared to females, and dorsal fin lengths increased within 10 days of protogynous (female to male) sex change. Epaxial and hypaxial are trunk muscles that power all movements through association with all limb muscles in fish. We conducted two separate studies to examine sex steroid levels in skeletal muscles of fish housed either in larger open communities, or in smaller, isolated groups. In fish from community groups, supracarinalis muscles in males had significantly higher levels of 11-ketotestosterone and lower estradiol compared to females, while isolated groups had no sex differences. In fish from isolated groups, only the epaxial and hypaxial muscles had plastic endocrine responses such that within only 5 days of transition, protogynous fish had significantly higher 11-ketotestosterone compared to stable males and intermediate estradiol compared to stable males and females. Overall, endocrine changes preceded morphometric changes and exhibited plasticity across different environments and life history stages. These results support the presence of many traits, from external morphological features to the cryptic endocrine level, that contribute to the occurrence of multiple morphs across life history stages in *L. dalli.* The integration of these phenotypes is important to understand the mechanisms that regulate the functional performance of behavioral displays in specific social contexts.

## Introduction

Phenotypic plasticity can manifest at the whole-organism level through a spectacular display of heightened states of behavior such as aggression and courtship that only occur under appropriate environmental contexts to maximize survivorship and reproductive success (Nilsen et al. 2004; Pradhan et al. 2015a; Schlinger et al. 2018). Sexually selected morphological traits such as exaggerated color, shape, or size of body parts play a role in sexual size dimorphism of birds such as manakins (Shogren et al. 2022), or sexual dimorphism in deer antlers (Gadgil 1972), are often amplified during performance of aggressive or courtship displays (Moore 1990). These displays often require whole body coordination through physiological activation of the neuromuscular system (Schlinger et al. 2013; Fuxjager et al. 2017). Morphological structure and function relationships are generally organized through exposure to steroid hormones at key moments during development and activated throughout the lifespan of an individual, contributing to sexually heteromorphic (Smiley et al. 2024) or dimorphic phenotype and behavior (Phoenix et al. 1959; Beach 1976). Hormones regulate phenotypic changes at different life history stages that range from temporary to permanent and affect morphology, behavior, and physiology (Bókony et al. 2008; Todd et al. 2019). The proximate mechanisms by which phenotypic flexibility is expressed in the overall display of behavior through the elaboration of particular morphological features, is highly context-dependent and likely to involve crosstalk across multiple organ systems.

Steroid hormone signaling mediates the expression of sexually dimorphic reproductive behavior in vertebrates by activating both central and peripheral neural circuits that innervate muscle targets (Fuxjager et al. 2017; Schuppe et al. 2017; Schlinger et al. 2018). Local production of steroid levels within tissues allows for spatial regulation of expression of phenotypes (Schmidt et al. 2008; Pradhan et al. 2015a). Androgens and estrogens are tightly correlated with aggression, courtship, fecundity, and many secondary sexual characteristics (Phoenix et al. 1959; Beach 1976; Hutchison and Steimer 1985; Tomaszyski et al. 2006; Remage-Healey et al. 2008; and Pradhan et al. 2010). Testosterone (T) is a major androgen for male vertebrates and is strongly associated with courtship behaviors and pair bonding (Marler et al. 1988; Adkins-Regan 2012). Androgen receptor (AR) activation and expression directly affects the extent to which T can alter the behavior of an organism including muscle tissue that support reproductive functions (Zuloaga et al. 2008), such as copulation in rodents (Bodo and Rissman 2008), elaborate courtship displays involving wing snapping in golden-collared manakins (Fuxjager et al. 2012; Schlinger et al. 2013), preparation for migratory flight in white-crowned sparrows (Pradhan et al. 2019b), hindlimb gestural displays and sexual clasping in frogs (Mangiamele et al. 2016), vocal muscle humming for sexual attraction in midshipman fish (Bass and Remage-Healey 2008), and courtship jerks in bluebanded gobies (Schuppe et al. 2017).

**Fig. 1:**
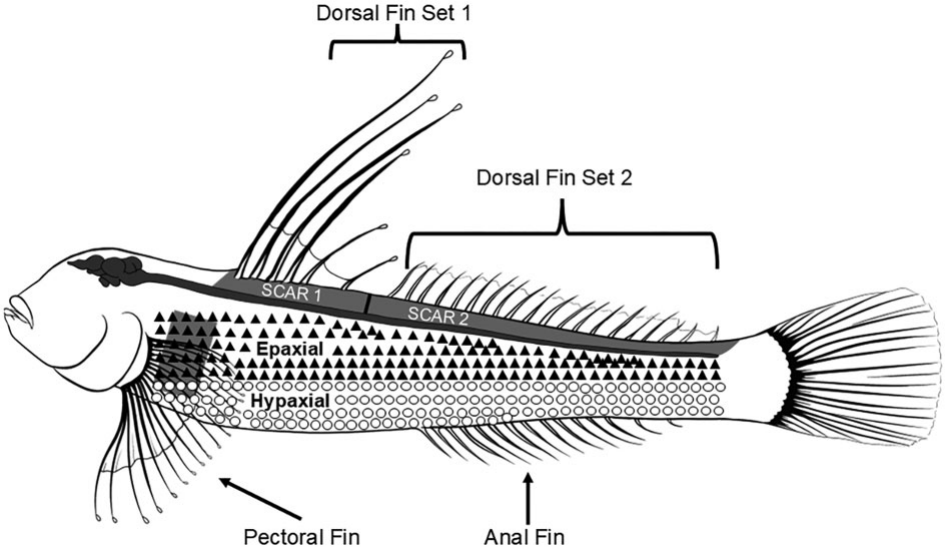
Diagram depicting a generalized *Lythrypnus dalli* showing different fins and muscles. Gray areas represent portions of muscle sampled. Triangles show the estimate of the epaxial muscle, while circles show the estimate of the hypaxial muscle that comprise the trunk. All the limbs attach with different portions of the epaxial and hypaxial muscles. All locations are estimates as the exact separation between the epaxial and hypaxial muscles is not known. Note: Dorsal Fin set 1 has fin rays of different lengths which can be individually manipulated by these fish. The first, second, or third fin ray can be longer and varies by each individual fish. Dorsal fin set two has fin rays of similar lengths and cannot be controlled individually.

Bluebanded gobies (*Lythrypnus dalli*) are teleost fish that inhabit the Pacific Ocean ranging from the coast of southern California to the Baja peninsula of Mexico. These are serial bi-directionally sex changing fish that live in a harem-like social structure consisting of one male and several females (St Mary 1997; Reavis and Grober 1999). External genitalia are reliable indicators of the “end point” active sexual state that can ensure appropriate gamete delivery, such that females have a rounded or flattened genital papilla (GP), with a length to width ratio <1.2 and males have a longer GP which is pointy at the end, with a ratio >1.8; also see (Schuppe et al. 2016). While juveniles who have recently settled onto reefs have undifferentiated GP, adult fish with pointy GP and an intermediate ratio are likely transforming to male. While males, mini males, and females externally appear monomorphic in coloration (Drilling and Grober 2005; Pradhan et al. 2015a), subtle differences in morphological characteristics have not been evaluated. For example, while dominant males are physically larger than females (St. Mary 1993), detailed landmarks have not been established for geometric or morphometric analyses in *L. dalli* as in other species (Keeley et al. 2021). Further, body size is not the only factor and multiple variables likely exist for establishing behavioral dominance (White et al. 2023) and mate choice. Additional differences in external anatomical features, such as fins and body shape might exist (author preliminary results), and might further contribute to the overall body structure but these have not been comprehensively evaluated. Male *L. dalli* perform a courtship display called the “jerk” swim that involves flaring of all sets of fins while swimming using rapid, jerky movements (Pradhan et al. 2015c; Schuppe et al. 2017). Dorsal fins are sets of unpaired appendages that run medially along the body (Fig. 1). Like other fish species (Helfman et al. 2009) both male and female *L. dalli* frequently use their dorsal fins for aggressive displays by increasing amplitude to a “fin raise” position during hierarchy formation (author unpublished results). While dorsal fin raise displays have not been previously described for *L. dalli*, one hypothesis is that a larger fish with longer dorsal fins can amplify the effect of a dorsal fin raise display, especially when two fish confront head-on and only these median fins are in view, thus aiding in establishing dominance. As a first step to bridge these gaps in knowledge, we evaluated dorsal fin morphometrics across wild-caught *L. dalli* at different life history stages.

The supracarinalis (SCAR), a muscle that attaches to two sets of spiny dorsal fins and controls movement of dorsal fins, runs parallel to the spinal cord (Fig. 1). While microscopic details of SCAR muscle fibers have not been characterized, there are sex differences in endocrine biomolecules at the histological protein expression level (Schuppe et al. 2017). Compared to females, male *L. dalli* have higher levels of AR expression and 11β-hydroxysteroid dehydrogenase Type 2 (11β-HSD2), an enzyme that synthesizes 11-ketotestosterone (11-KT), within the first region of their supracarinalis (SCAR 1) muscle that is associated with the first set of dorsal fins (Fig. 1) (Schuppe et al. 2017). Further, there is no sex difference in AR or 11β-HSD2 expression in the epaxial and hypaxial muscles (E/H; trunk muscles) in the portion associated with the pectoral fins (Schuppe et al. 2017). Higher levels of AR expression in the SCAR 1 is also associated with higher rates of courtship jerks displayed by males (Schuppe et al. 2017). While a receptor specific to 11-KT has yet to be discovered in teleost fish, it is commonly assumed that activating effects of 11-KT will be initiated via binding AR (Borg 1994; Olsson et al. 2005; Yazawa et al. 2008) and like dihydrotestosterone, 11-KT binds to the AR with a much higher affinity than T. Preliminary work shows that female *L. dalli* have significantly lower mRNA expression of AR across several tissues (A. Symanietz and D. Pradhan, unpublished results), while it is well known that there are no differences in systemic 11-KT levels in the top ranking males and alpha females (Lorenzi et al 2008; White et al 2023). Rather than systemic measures, local levels of steroids within specific tissues provide a deeper insight into spatial regulation mechanisms, as there can be differences both in the different types of steroids and types of tissues. Interestingly, there is also no sex difference in caudal muscle or gonadal T and 11-KT levels within *L. dalli*; however, 17β-estradiol (E_2_) is higher in both caudal muscle and gonads of females compared to males (Lorenzi et al. 2012). In this previous study, the muscle sample was taken from the caudal portion and cut parallel to the caudal peduncle, containing attachments to SCAR 2 (supracarinalis muscle associated with the second set of dorsal fins), epaxial, and hypaxial muscles. Here, we focused on assessing SCAR separately from E/H muscles because these muscles connect to different fin types and physiological differences in local steroid levels might help understand how complex and intricate fin movements pertaining to reproductive behavior are regulated.

Over the past few decades, fish systems have been investigated to understand biomechanics of propulsion and locomotion, but there is very little information on how complex behaviors are controlled and integrated from a morphological to physiological level (Flammang 2014). To fill the gaps in knowledge, we focused on examining peripheral aspects of phenotypic differences in dorsal fin morphology and muscle steroid hormones of males, females, and transitioning fish (juvenile and adults). In study 1, we asked if there were differences in the longest dorsal fin ray length (DFL) to standard length (SL) ratio across males, females, and juveniles that were housed in large mixed sex community tanks, in densities similar to densely populated regions along reefs. We hypothesized that males would have a larger DFL/SL ratio than that of both females and juveniles, as a result of phenotypic plasticity stemming from sexual selection pressures. Given that both the first and second set of dorsal fins attach to the SCAR, in study 2, we asked if there are differences in T, 11-KT, and E_2_ concentrations within the first and second regions of the SCAR muscle among males, females, and juveniles (Fig. 1). We hypothesized that there are differences in T, 11-KT, and E_2_ concentrations between both regions of the SCAR musculature across morphs in specific life-history stages. In study 3a, we investigated isolated social groups and first asked if there were any differences in T, 11-KT, and E_2_ concentrations within the SCAR 1 and E/H muscles of fish that were at 5 days of protogynous (female to male) sex change (T5) compared to males and females from stable groups. We hypothesized that the DFL would begin to grow in length and that there would be a large spike in 11-KT concentration within muscles after protogynous sex-change was induced, given that 11-KT transiently increases in both brain and gonad during this process. In study 3b, we investigated changes in sexual plasticity of dorsal fin morphology during the first 10 days of protogynous sex change in fish from isolated groups.

## Methods

### Field protocol

Distribution of *L. dalli* ranges from Morro Bay, CA, USA, down to the Gulf of California, Mexico (Miller and Lea 1972). Wild *L. dalli* exist in relatively large mixed sex groups containing individuals at multiple life-stages. Multiple fish were captured in hand nets using SCUBA diving off the coast of Santa Catalina Island, CA, USA (California Department of Fish and Wildlife Permit ID: S192610005-19271-001). Captured fish were stored in Nalgene bottles for transport to the boat, where they were then kept in a large bucket with fresh seawater out of direct sunlight. Fish were then taken to the laboratory at the University of Southern California Wrigley Marine Science Institute. All fish were put into a community sea table containing 100–150 fish, before they could be processed or put into study groups.

### Study 1—Morphometrics of fish from community groups

Within 24–48 h of being brought into the lab, using a hand net, fish were randomly collected from two community sea tables that had multiple harems and randomly placed nesting sites. These nesting sites consisted of varied environmental enrichment such as abalone shells, empty sea urchin tests, rocks, and additional PVC tubes that were added as nesting sites. Additional bycatch invertebrates such as gastropods, shrimp, nudibranchs, and sea stars were also in these community tanks. Fish in the sea tables were continually replenished with new catch across the breeding season. Fish were observed to be interacting with each other socially before collections, but no detailed behavioral observations were made due to the difficulty of individually identifying and tracking focal fish in the large group setting (Rodgers et al. 2005). Before processing, fish were anesthetized with Tricaine-S in fresh seawater. Using a dissecting microscope, sex was determined based upon the GP morphology and categorized as male, female, or juvenile individuals (St Mary 1993; Schuppe et al. 2016; Solomon-Lane et al. 2016). Using Vernier Calipers, SL and DFL of the longest dorsal fin ray were measured (Fig. 1), then fish were weighed, and a digital photograph of GP was taken using an external camera (Moticam) mounted to the microscope. Images were transferred to a laptop computer (Motic Images Software system running on a MacBook). A total of 398 individuals were used to establish morphometric and sexual dimorphism within *L. dalli*, males: *n* = 100, females: *n* = 283, juveniles: *n* = 15.

### Study 2—Sex steroids in muscles of fish from community groups

We investigated two sections of the SCAR muscle (Fig. 1). Tissue mass was taken by placing the sample on sterile aluminum foil squares on a DeltaRange^©^ XP105 microbalance and homogenized in 350 µL 0.1 M borate buffer closely following previous established protocols (Lorenzi et al. 2012; Pradhan et al. 2014a). Concentrations of sex steroids within both SCAR regions and E/H musculature were measured using Cayman Chemical T, E_2_, and 11-KT enzymeimmunoassay kits following all Cayman Chemical recommended protocols as in previous studies (Pradhan et al. 2014a; White et al. 2023).

### Dissection of supracarinalis

The fish was oriented so that it was laying on its ventral side. One dissecting pin was placed in the caudal fin and in each of the pectoral fins. Using micro scissors, the dorsal fins were cut off at the fin attachment points, and a transverse cut was made on the dorsal side that ran parallel with the 1st band of body markings, approximately 2 mm deep and 1–2 mm laterally from the dorsal midline. Another 2 mm deep cut was made, where the second set of dorsal fins ended. Then, using the micro knife, scales were carefully removed from the section of tissue. Last, using micro scissors, a 70° angle cut was made, running the entire length of the dorsal midline, on each side 1 mm from the dorsal fins and ∼2 mm deep. As a landmark, the cut was deep enough to pass just above the spine of the vertebrae, which provided resistance. The final tissue sample was triangular and long in shape; SCAR 1 was associated with the first set of dorsal fins while SCAR 2 was associated with the second set of dorsal fins. Once the SCAR muscle was removed, it was immediately frozen on dry ice and then placed in a microcentrifuge tube before storing at −80°C.

### Dissection of epaxial and hypaxial muscles

After morphological assessments, a subset of fish was euthanized with Tricaine-S and used for this study (*n* = 26). Given that these fish naturally have bands along their bodies, we used these as landmarks for our dissections. Each fish was laid down on its lateral side, randomly turned to the left or the right side, with a dissection pin placed at the base of the caudal fin and one in the gills. Using a micro knife, a square cut was made starting from the area of the pectoral fin attachment, with one cut running parallel to the dorsal midline (from the 3rd to the 4th band) and another cut running dorsal to ventral, joining the other two cuts, and 2 mm deep. Scales were removed from muscle tissue using the micro knife. Once the epaxial and hypaxial muscles were removed, these were immediately frozen on dry ice and then placed in a microcentrifuge tube before storing at −80°C. At the end of the field season, both E/H and SCAR tissue samples were transported on dry ice to Idaho State University and stored at −80°C for steroid measurements.

### Study 3—Sex steroids in muscles and fin morphometrics of fish from isolated groups

Fish were taken from the community sea table and after morphological assessments, placed in social groups. Social groups either consisted of 1 male and 2 females or of 3 females (males: *n* = 6, females: *n* = 6, transitional: *n* = 6); the latter type of group created a permissive social environment for protogynous sex change for the most dominant female. These combinations allowed for behavioral and morphological measurements in a controlled environment. In both cases, we created groups where the largest fish was at least 3 mm larger than the second, and the second fish was at least 3 mm larger than the smallest, following previous methods (Black et al. 2005a; Pradhan et al. 2014a). Differences in banding pattern combinations of fish provide an additional method to identify individual fish in small community groups and easily track behavior.

All groups developed a stable social hierarchy before any perturbation occurred, which we verified using social behavior observations that were similar to multiple past studies (data not shown here for brevity). In the case of all female groups, once this hierarchy was established, the most dominant female, here on, the transitioning fish, immediately began the protogynous sex change process (Rodgers et al. 2005). For study 3a, a subset of transitioning fish (*n* = 6) were collected at 5 days following the induced protogynous sex-change event (T5) and used for morphometric and muscle steroid hormone analysis. Nesting (stable) males (*n* = 6) and stable females (*n* = 6) not undergoing sex change were also used to compare with T5 fish. Fish were euthanized with an overdose of Tricaine-S dissolved in sea water, morphometric measures were taken, and then fish were immediately dissected to retrieve the SCAR 1 and E/H muscles. Two transitioning fish from this study had ick, and therefore we did not take steroid measurements, but included these individuals within the morphometric measures. For study 3b, another subset of *n* = 6 groups of 3 females each was set up to measure morphometric changes in DFL in transitioning fish across 5 days and 10 days following the induced protogynous sex change event.

### Statistical analysis

All data were checked for normal distributions where zero values were not present and analyzed using Graph Pad Prism v10.6.1 statistical software. In study 1, we performed a simple linear regression to compare DFL vs. SL, a one-way analysis of variance (ANOVA) with a Tukey *post-hoc* analysis to compare DFL/SL ratio of males, females, and juveniles from community groups. In study 2, we used multiple one-way ANOVAs with Tukey *post-hoc* analyses to compare T, 11-KT, and E_2_ in SCAR 1 and SCAR 2 of male, female, and juvenile *L. dalli* from community tanks. In study 3, we compared 11-KT and E_2_ in SCAR 1 and E/H muscles of males, females, and transitioning fish involved in study isolated groups using multiple one-way ANOVAs and Tukey *post-hoc* analyses. Further, we used RM-ANOVA with Tukey *post-hoc* analysis to investigate DFL/SL changes over time in transitioning fishes from isolated groups for study 3b. If no steroid was detectable in a sample, then those samples values were set to zero and included in all statistical analyses as to indicate a lack of steroid signal in those respective tissues. All data represented here are shown as mean ± SEM with α = 0.05, excluding the ratio analyses.

## Results

### Study 1—Morphometrics in community groups

**Fig. 2:**
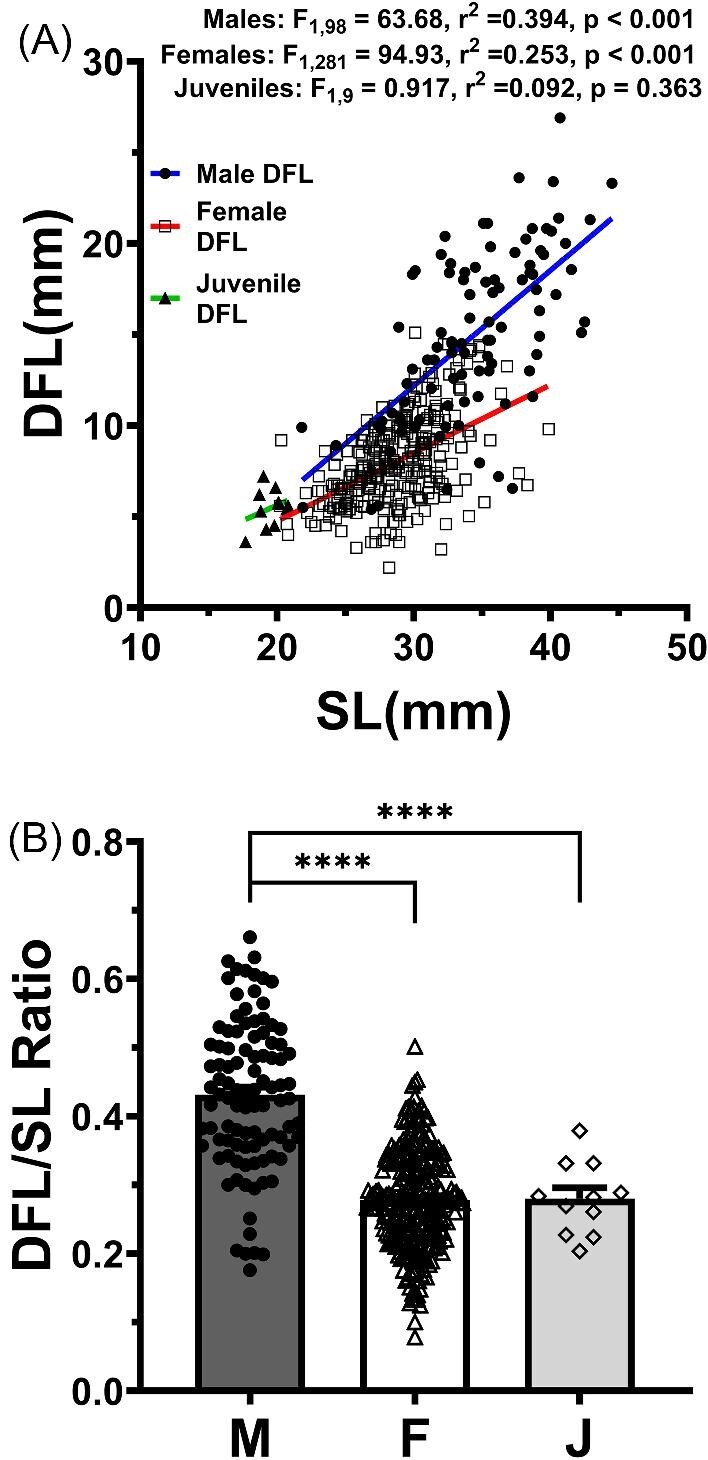
(A) Simple linear regression of DFL and SL of male, female, and juvenile *L. dalli*. Male and female lines are different from each other. (B) A standardized morphometric comparison used to evaluate phenotypes in multiple morphs across life history stages, such as among males, females, and juveniles. Males (M): *n* = 100; Females (F): *n* = 283, Juveniles (J): *n* = 11. ****P < 0.0001.

A simple linear regression revealed that there was a significant relationship between DFL and SL in males (*r*^2^ = 0.394, *P* < 0.001; Fig. 2A) and females (*r*^2^ = 0.253, *P* < 0.001) but not in juveniles (*r*^2^ = 0.092, *P* < 0.363). A one-way ANOVA showed that overall, there was a significant difference across fish from different life history stages (*F*_2,390_ = 130.6, *P* < 0.001; Fig. 2b). Specifically, a Tukey *post-hoc* analysis indicated that the standardized ratio of DFL/SL in males were significantly different from both females (*P* < 0.001) and juveniles (*P* < 0.001), but that females and juveniles were not different (*P* = 0.996).

### Study 2—Sex steroids in muscles of fish from community groups

**Fig. 3:**
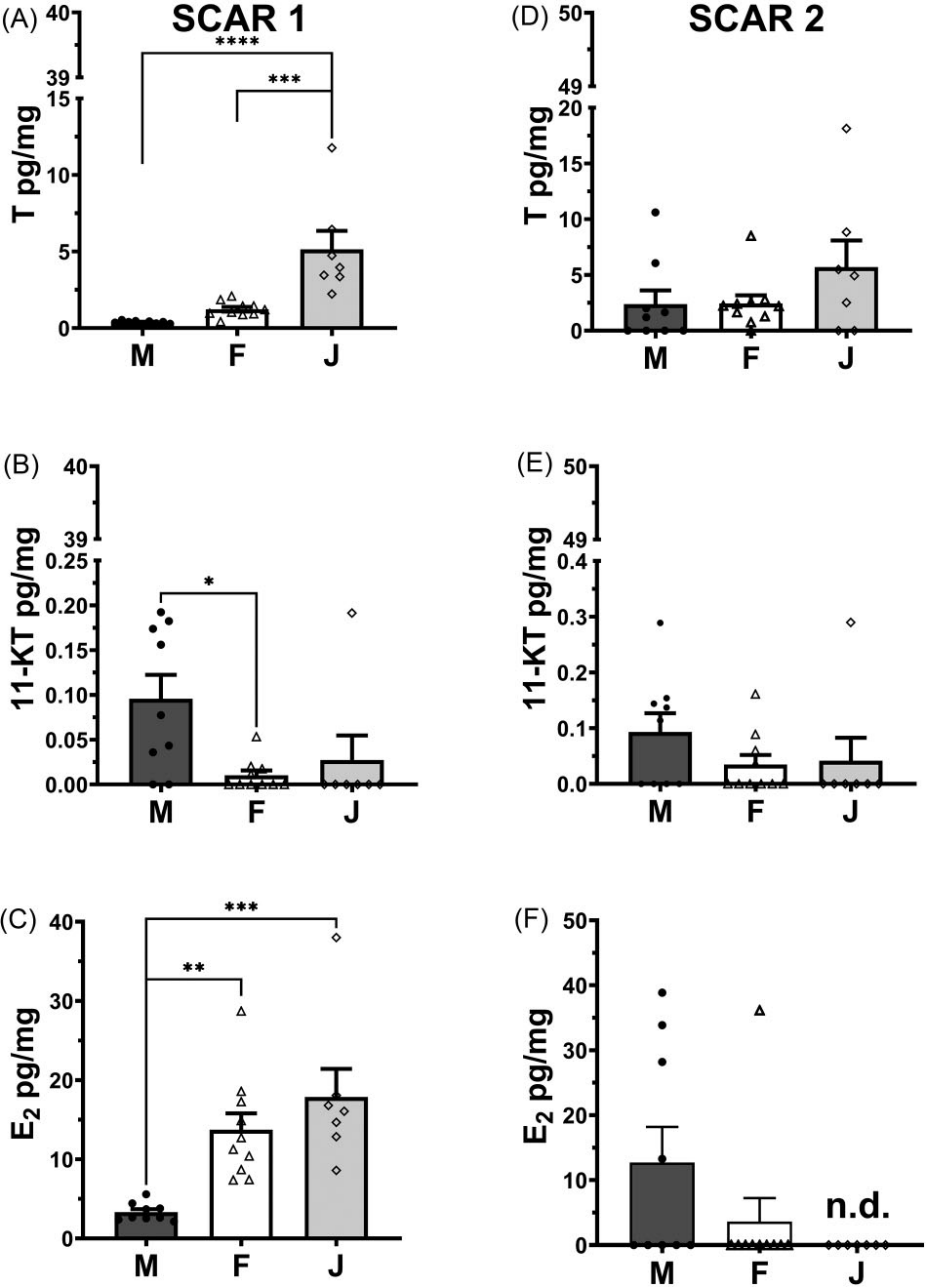
Comparison of testosterone (T, A and D), 11-Ketotestosterone (11-KT, B and E), and 17β-estradiol (E_2_, C and F) within the supracarinalis region 1 and 2 (SCAR 1 and SCAR 2) tissue of males (M), females (F), and juveniles (J) from a community tank. Males: *n* = 9; females: *n* = 10; juveniles: *n* = 7. * indicates significance at *P* = 0.05, **P = 0.01, ***P = 0.001, ****P < 0.0001, and n.d. indicates no steroids were detected.

Analysis of SCAR 1 indicated that there were significant differences in all three hormones across fish from different life history stages. A one-way ANOVA showed T in SCAR 1 to be significant (*F*_2,23 _= 17.72, *P* < 0.001; Fig. 3A). A Tukey *post-hoc* indicated that juveniles had higher T than both males (*P* < 0.001) and females (*P* < 0.001), but males and females were not different (*P* = 0.516). For 11-KT in SCAR 1, a one-way ANOVA was significant (*F*_2,23_ = 5.022; *P* = 0.016; Fig. 3B). A Tukey *post-hoc* analysis indicated those males had higher 11-KT than females (*P* = 0.015), but that males were not different from juveniles (*P* = 0.089), while females and juveniles (*P* = 0.837) were similar. However, it should be noted that the result between males and juveniles is driven by one juvenile data point and that the remaining juveniles had no detectable 11-KT in their SCAR 1 tissue. For E_2_ in SCAR 1, a one-way ANOVA was significant (*F*_2,23 _= 11.58, *P* < 0.001; Fig. 3C). Specifically, a Tukey *post-hoc* indicated that males had significantly lower E_2_ from females (*P* = 0.004) and juveniles (*P* < 0.001), but that females and juveniles (*P* = 0.399) were similar. Analysis of SCAR 2 indicated that there were no differences in T (*F*_2,23 _= 1.615, *P* = 0.221; Fig. 3D), 11-KT (*F*_2,23 _= 1.171, *P* = 0.328; Fig. 3E), and E_2_ (*t* = 1.406, *df* = 17, *P* = 0.178; Fig. 3F) concentrations among males, females, and juveniles. Additionally, it should be noted that the results of SCAR 2 were driven by a small number of individual fish where steroids were detectable. This was especially prevalent in the case of E_2_ within SCAR 2 where no detectable steroid was found in any juveniles. This is in converse to SCAR 1, where steroid concentrations were readily detectable among all sexes in most samples.

### Study 3—Sex steroids in muscles and fin morphometrics of fish from isolated groups

**Fig. 4:**
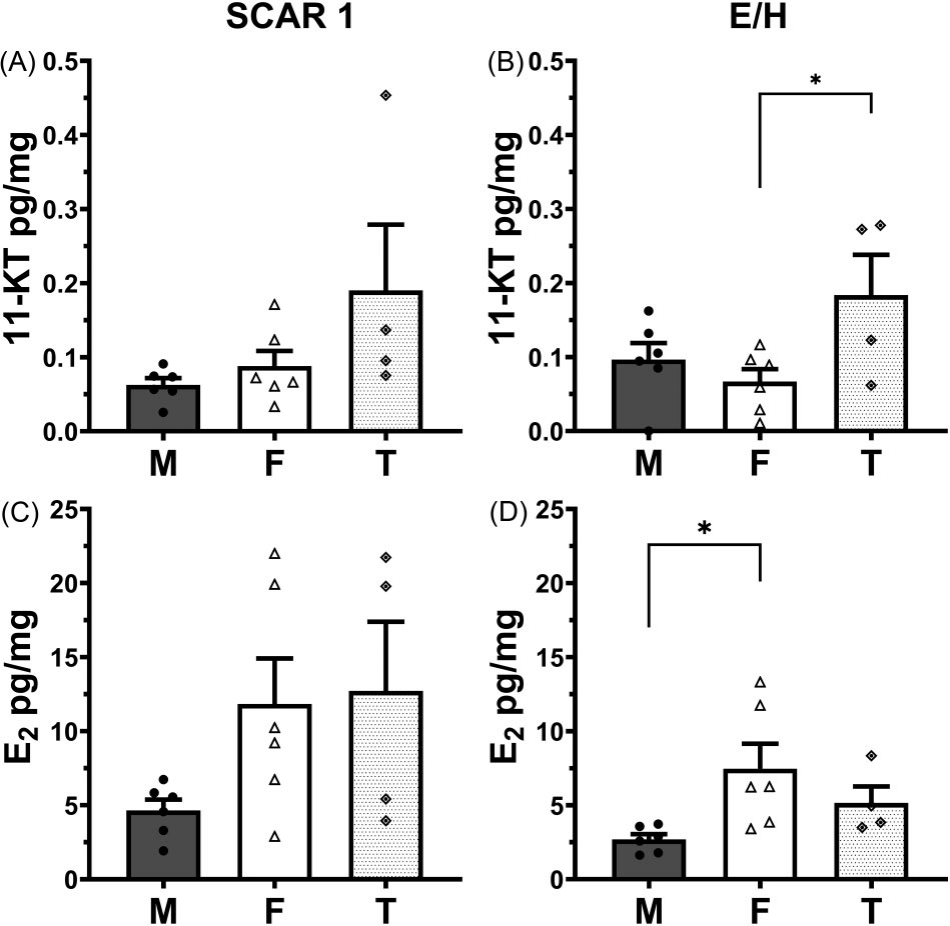
Concentrations of 11-ketotestoterone (11-KT, A and B) and 17β-estradiol (E_2_, C and D) in the supracarinalis 1 (SCAR 1) and epaxial/hypaxial (E/H) tissues of males (M) and females (F) in stable groups, and female to male transitioning (T5) fish from isolated social groups. Males: *n* = 6; Females: *n* = 6; Transitioning: *n* = 4. *P = 0.05.

A one-way ANOVA revealed that while SCAR 1 had similar 11-KT concentrations in males, females, and transitioning fish (*F*_2,13_ = 2.467, *P* = 0.124; Fig. 4A), E/H had significantly different 11-KT across these morphs (*F*_2,13_ = 3.738, *P* = 0.052; Fig. 4B). Specifically, a Tukey *post-hoc* analysis indicated that females had lower 11-KT than juveniles (*P* = 0.046), but males and females (*P* = 0.732) and males and juveniles (*P* = 0.150) were similar. Again, SCAR 1 showed similar E_2_ concentrations in males, females, and transitioning fish (*F*_2,13_ = 2.490, *P* = 0.122; Fig. 4C). However, for E/H, E_2_ concentrations were significantly different (*F*_2,13_ = 4.299, *P* = 0.037; Fig. 4D) such that females had a higher E_2_ levels than males (*P* = 0.029) but not transitioning fishes (*P* = 0.436). Males and juveniles had similar E_2_ levels in their E/H (*P* = 0.393). The protogynous transitioning fish, T5, did not have changes in their DFL/SL (*P* = 0.449) or length to width ratio of their GP compared to their pre-study GP ratio (*P* = 0.617; Table 1).

**Table 1:** Data regarding the change in GP of transitioning fishes tracked from day 0 (D0) to day 5 (D5) during protogynous sex-change; *n* = 4.

Sex	D0 Initial GP ratio (±SEM)	D5 GP ratio (±SEM)	*P*-value
Transitioning	0.960 (**±**0.230)	1.180 (**±**0.090)	0.617

**Fig. 5:**
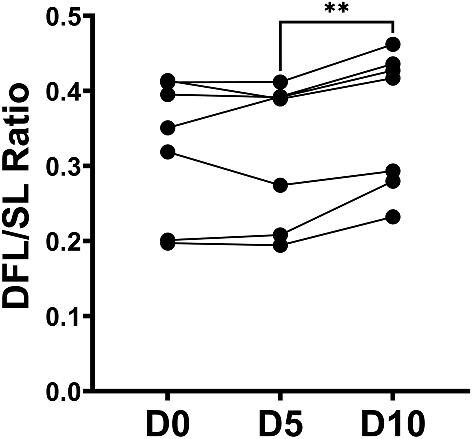
Differences in standardized DFL/SL ratio of transitioning fish from the initiation (D0) of protogynous sex-change to 5 days (D5) and 10 days (D10) after the initiation of sex change began. *n* = 7, **P = 0.01.

We also found changes in the DFL/SL ratio of transitioning fish from study 3b using repeated measures one-way ANOVA (*F*_1.135_,_6.810_ = 8.374, *P* = 0.022; Fig. 5). Specifically, transitioning fish had an increase in DFL/SL ratio from day 5 to day 10 after protogynous sex-change was initiated (*P* = 0.002) but no changes between day 0 and day 5 (*P* = 0.928) or day 0 and day 10 (*P* = 0.103) were detected.

## Discussion

Across the lifespan, many species experience growth associated with changes in form and structure of limbs. The association of the endocrine system in orchestrating morphological changes at both microstructural and macrostructural levels can be explored in species that experience drastic and complex life history changes. Here, we studied such hormone-morphology differences across different life stages in a sexually plastic fish, *L. dalli*. First, we compared limb (dorsal fin) size and body sizes across juveniles, adult males, and adult females and found both differences and a continuum in morphometrics during adulthood, identifying the existence of multiple morphs that have this sexually fluid characteristic. Next, we assessed steroid hormone levels across specific muscle tissues that are associated with dorsal fins and with performance of complex behaviors. We found differences based on both the life stage and social contexts of housing fish. Our results provide novel insights into the plasticity of muscular endocrine pathways at different life stages and during natural sex change. Moreover, we found changes in length of the dorsal fins associated with protogynous sex change within only 10 days, further underscoring the manifestation of multiple changes at the whole organism level. While most previous studies investigating sex change have focused on reproductive allocation and some on the brain–behavior relationships, this study extends our understanding to include limbs and muscles—anatomical sites that are not traditionally investigated.

### Overlap in size in dorsal fin length across adult morphs

The distribution pattern of DFL across body size (SL) reveals that these characteristics are on a continuum, such that there is a great deal of overlap between males and females. In fact, while 45.54% males, had dramatically longer dorsal fins compared to females, more than half the males had similar or even smaller DFL than females (Fig. 2A). One explanation for this phenomenon is that the role of body size contributing to a dominant social status is only relative to other fish in the group. The dorsal fin is used by all fish for steering movement in specific directions regardless of social status, and for communication via fin raise displays (author, in preparation). Whether the selection pressures contributing to the length of the fins are related to sex, are presently unresolved and will be investigated through assessment of mechanics of movement in future.

Our novel finding that dorsal fin length increased within only 10 days of protogynous sex change in study 3b (Fig. 5) supports the hypothesis that DFL is sexually selected. We added this study to test for a longer timepoint at 10 days because T5 fish in study 3a did not have any changes in their DFL/SL. Similar to previous studies, the GP ratio (Table 1) for those fish had also not changed as 5 days might be too early to detect reproductive-level changes. While the rearrangement of reproductive organs during the long-term sex change are well known (Manabe et al. 2013; Sunobe et al. 2017), this work demonstrates plasticity of a body appendage, rather than just body size at the macro level in *L. dalli* (Rodgers et al. 2007). These phenomena of sexual dimorphism and morphogenesis of dorsal fins during protogynous sex change but not during protandrous sex change in another closely related species, *L. pulchellus* (Muñoz-Arroyo et al. 2019), indicating unidirectional plasticity in fin growth. However, that study had not standardized the DFL measures relative to SL, providing a slightly different perspective in understanding the application of this morphometric to the organismal level. Whether the longer dorsal fins in males confer a reproductive advantage is yet to be unraveled; however, further analysis of male specific behaviors that use dorsal fins, such as courtship jerk swims and parenting (Pradhan et al. 2015c; Schuppe et al. 2017), might provide a more detailed understanding toward the utility of dorsal fins. The present study only focused on the longest ray of the dorsal fin, but additional morphometric details and biomechanics will provide further insight.

Juvenile *L. dalli* concentrate at the lower end of the spectrum, with very little variation in the distribution of the SL versus DFL across all fish (Fig. 2A). While juvenile fish do not have a GP that clearly identifies with specific sexual morphs, they generally lean towards being more female-like due to their smaller SL at this early life history stage. As they have social group dependent sex determination, group composition is likely to contribute to the further expression of GP morphology (Solomon-Lane et al. 2016). Moreover, at this small size, juvenile *L. dalli* have similar DFL/SL as females, which is consistent with the finding that most juveniles have female-typical GP and gonad morphology (Solomon-Lane et al. 2016). Further investigation of plasticity in DFL of juveniles as they integrate into social groups and emerge as adults will provide a deeper understanding of the external social environment contributing to the size of dorsal fins during early development. Given that these fish are bidirectionally sexually plastic throughout their life, there are likely to be many nuances associated with mechanisms associated with these phenotypes.

### Importance of SCAR1 as a prominent site of sex steroid concentration across morphs

There were many differences in patterns of all three hormones across the two sets of SCAR in study 2 (fish that were housed in community tanks). In SCAR 1, while juveniles had dramatically higher T levels than both males and females (Fig. 3A), 11-KT levels were highest in males compared to females and juveniles. This latter result is in line with previous findings, that males have higher levels of 11β-HSD2 and AR expression in muscles compared to females (Schuppe et al. 2017). Together, those data indicate that the enzyme necessary for 11-KT synthesis and signaling are all expressed in the SCAR 1 region of males. Combined with the present work that males also had significantly lower levels of E_2_ than females and juveniles suggest that the steroidogenic pathway might be channeled in an androgenic direction in male muscles versus an estrogenic direction in juveniles and females. Future studies will investigate whether aromatase enzyme expression and activity are higher in the SCAR 1 region of females and juveniles, which would allow for local conversion of T to E_2_. This is in stark contrast to the SCAR 2 region, where most females and juveniles had no detectable E_2_ or 11-KT. Given that there was little to non-detectable steroid concentrations in SCAR 2, this region is expected to be of lesser importance for future investigations.

Whether the differences in estrogenic versus androgenic pathways are byproducts of a lower social rank of these morphs compared to dominant males warrants further investigation. Alternatively, the higher 11-KT levels in SCAR 1 might be associated with males holding nesting territories; these males were actively displaying their fins at other fish in their vicinity at very high rates (author, personal observation) as these fish constantly compete with other fish in a community social environment for the nesting territory. Thus, these community groups are a high competition environment, and 11-KT levels are likely representative of a social challenge in these fish, that is, the “challenge hypothesis” (Wingfield et al. 1990). The transient increases in 11-KT during social hierarchy establishment occurs in the brains of *L. dalli* as early as 30 min and remains elevated after 24 h (Lorenzi et al. 2012; White et al. 2023). Undoubtedly, muscles are engaged in the production and performance of specific aggressive displays and thus could be hypothesized to have independent local activation of the endocrine signaling molecules that control the timely expression of these behaviors. Indeed, muscles are capable of independent steroid hormone production and signaling regulation in other reproductive behavior contexts such as courtship in birds and frogs (Girgenrath and Marsh 2003; Fuxjager et al. 2012, 2016; Eaton et al. 2018; Schuppe et al. 2022). In future, this hypothesis can be tested in *L. dalli* through a combination of steroidogenic enzyme activity assays in muscles and using muscle intramuscular pharmacological manipulations of endocrine network molecules (Munley et al. 2022; Smiley et al. 2024).

### Physiological significance of differences in E/H sex steroids across morphs in isolated groups

In the epaxial and hypaxial muscles, the transitioning fish had significantly elevated 11-KT levels compared to stable males and females (Fig. 4B). In contrast to fish from community groups, stable males and females in isolated groups had similar 11-KT levels in SCAR 1, similar to previous studies investigating systemic 11-KT levels (waterborne levels) (Lorenzi et al. 2008; Pradhan et al. 2015b; White et al. 2023) as well as brain, caudal muscle and gonads (Lorenzi et al. 2012; Pradhan et al. 2014b; Pradhan et al. 2015b); and while 11-KT was slightly elevated in protogynous transitioning fish, it was not significantly higher (Fig. 4A). The male and female morphs in these isolated groups had attained a stable hierarchy at the time the muscle tissues were collected; however, the transitioning fish were likely showing the transient increases in 11-KT levels that are likely representative of the social challenge and/or organogenesis related to protogynous sex change (Lorenzi et al. 2012; White et al. 2023). This may indicate that there are significant differences in concentration of steroids in muscle tissue and systemic circulation. Here, we focused on muscle tissue alone because we were concerned more with steroid concentrations in muscle at a specific time. Unfortunately, plasma samples cannot be taken from *L. dalli* due to their small size leading to waterborne hormones being the standard. This creates dissonance between systemic and tissue level samples because muscle tissues serve as a snapshot of the steroid concentration, whereas waterborne hormones need to be collected over an interval of an hour, leading to possible changes in concentration levels during this time.

Another study that focused on AR and 11β-HSD2 found basal levels of both these androgen-signaling molecules in stable males and females. In several past studies involving *L. dalli*, 11-KT has been found to be elevated in both systemic circulation and tissues in transitioning fish or those experiencing short-term social challenges or males invested in parental care (Rodgers et al. 2006; Lorenzi et al. 2012; Pradhan et al. 2014a; White et al. 2023). Here, the higher local levels in the muscle (Figs. 3 and 4) might indicate the overuse of their appendages during whole body aggressive displays or increased movement patterns associated with establishing and guarding territories. Thus, the increased 11-KT in this muscle could be a product of overall increases in the E/H by virtue of its connection with other muscles during protogynous sex change in the T5 fish. This provides evidence for local production in muscles, regulated by steroidogenic enzymes such as 11β-HSD2, similar to what has been found in birds such as golden collard manakins (Fuxjager et al. 2016; Schuppe et al. 2022) and other migrating birds such as white crowned sparrows (Pradhan et al. 2019a, 2019b).

While SCAR 1 did not have significant differences in E_2_ levels across morphs from isolated groups, females had significantly elevated E_2_ levels compared to males in the E/H muscles (Fig. 4). Sex differences in E_2_ levels in *L. dalli*, with females having higher concentrations than males in systemic circulation and local tissues such as brain, gonad, and caudal muscles has been seen in multiple past studies (Lorenzi et al. 2008, 2012; White et al. 2023). The protogynous T5 fish had E_2_ levels that were between that of males and females, suggesting a “midway” phenotype. Even though T5 fish had established themselves at the top of the hierarchy, they are still typically undergoing organogenesis and there is likely to be a delay in detecting those cryptic changes at the endocrine level of analysis in muscles. In the brain, however, aromatase activity drops drastically over time in the early phases of sex change (Black et al. 2005b), indicating an endocrine switch. However, the time scale of those changes is variable depending on hierarchy resolution time of the conspecifics within a group (Black et al. 2005b). We predict that the precise timing of change would be challenging to detect at the peripheral gross muscle level. Additional changes at the microstructural anatomical level, coupled with gene-expression level changes would provide a more holistic endocrine network analysis during sex change.

### Integrating DF and SCAR muscles across morphs

Our preliminary analyses have revealed that the two sets of dorsal fins can change in orientation independently of each other. Our hypothesis is that the dorsal fins can be used by fish for a gesturing mode of communication, similar to their pectoral fins (Bass and Chagnaud 2012) and that these could be under local androgenic regulation. Given that juveniles also integrate into social groups and can demonstrate aggression, their first set of dorsal fins do maneuver; however, they are overall smaller, and their second set of dorsal fins associated with SCAR 2 is very difficult to see clearly. Other fin types associated with secondary sexual characteristics such as adipose fins (located posterior to dorsal fins) in the brown trout, *Salmo trutta fario*, also show differences across morphs based on age, such that older males have a greater adipose fin length than females and younger fish (Hisar et al. 2012). Moreover, older male *S. truta fario* adipose fins also have higher numbers of ARs than younger fish, also providing evidence for local androgen sensitivity (Hisar et al. 2012). Overall, in *L. dalli*, the range of sizes of the first set of dorsal fins and associated local levels of androgens suggest that SCAR 1 is associated with more variation in steroidogenesis associated with phenotypic differences across morphs.

### Limitations and significance

While this is the first demonstration of sex steroid levels sequestered within muscles, an associated weakness of our approach was that gross SCAR dissections were incredibly challenging due to the location and size of the muscle that are amplified due to the small SL of *L. dalli.* Thus, there might be additional surrounding tissue collected with the SCAR tissue, affecting the signal to noise ratio. However, the dissections were performed consistently within each study. Future studies will increase precision of these dissections by separating skin and fin insertion points that will be included more carefully. Measurement of the steroid hormones specifically, as these are products of enzyme activity and the molecules that bind receptors are important aspects of endocrine signaling networks (Munley et al. 2022). This study identifies both morphological plasticity and cryptic endocrine flexibility across life stages, social contexts, and adult sex transitions as a first step to understand the dynamic properties of muscles given that these organ-systems are representative of the fins to brain connection and engaged in the performance of specific aggressive behaviors. While the precise coupling of expression of behavior and endogenous signaling is challenging to unravel, our data are likely indicative of mechanisms that might promote tissue remodeling associated with multiple morphs in a single species, allowing an integrative organismal level assessment associated with dramatic life history transitions.

## Data Availability

All data will be made available upon request.
